# Combination of Ferulic Acid, Ligustrazine and Tetrahydropalmatine attenuates Epithelial-mesenchymal Transformation *via* Wnt/β-catenin Pathway in Endometriosis

**DOI:** 10.7150/ijbs.60167

**Published:** 2021-06-11

**Authors:** Chengling Zhang, Ying Zhang, Haiying Pan, Yi Tan, Qinghua Wei, Xueshan Dai, Jiahui Wei, Yi Chen

**Affiliations:** 1 College of Pharmaceutical Sciences & Chinese Medicine, Southwest University, Chongqing, China.; 2 Chongqing Key Laboratory of New Drug Screening from Traditional Chinese Medicine, Chongqing, China.; 3 Pharmacology of Chinese Materia Medica - the Key Discipline Constructed by the state Administration of Traditional Chinese Medicine, Chongqing, China.; 4 National Demonstration Center for Experimental Pharmacy Education (Southwest University), Chongqing, China.; 5 Sichuan Jinxin Women & Children Hospital, Chengdu 610066, China.

**Keywords:** Ferulic acid, Ligustrazine, Tetrahydropalmatine, Allograft endometriosis, Epithelial-mesenchymal transformation, Wnt/β-catenin pathway

## Abstract

Previously the potential therapeutic action of ferulic acid, ligustrazine and tetrahydropalmatine (FLT) are discovered with unclear mechanism in rat autograft endometriosis. However, the effect of FLT on endometrial cells and allograft endometriosis is still unclear. This study is designed to elucidate the influence of FLT on epithelial-mesenchymal transformation in allograft endometriosis and endometrium cells. *In vivo*, fluorescent xenogeneic endometriosis model was established. *In vitro*, invasion and metastasis were analyzed after treating FLT. Epithelial-mesenchymal transformation and Wnt/β-catenin pathway were inspected *in vitro* and *in vivo*. Activator or inhibitor of Wnt/β-catenin signaling was performed to inspect mechanism of epithelial-mesenchymal transformation. *In vivo*, FLT not only decreased fluorescent intensity and volume of ectopic lesion, but also ameliorated pathological morphology. E_2_ and PROG levels in serum were reduced by FLT. In endometrial cells, FLT significantly inhibited the invasion and metastasis. Meantime, epithelial-mesenchymal transformation was reversed, accompanied by suppression of Wnt/β-catenin pathway. In-depth study, activation of Wnt/β-catenin pathway lead to promotion of epithelial-mesenchymal transformation, which was reversed by FLT. FLT prevented fluorescent allograft endometriosis and endometrium cells, which was related to suppress epithelial-mesenchymal transformation through inactivating Wnt/β-catenin pathway. The findings disclose molecular mechanism of epithelial-mesenchymal transformation in endometriosis by FLT, and contribute to further application.

## Introduction

Epithelial-mesenchymal transformation is the cell process from epithelial to mesenchymal phenotype, then acquisition of invasion and metastasis. In epithelial-mesenchymal transformation, E-cadherin attenuates as one of the epithelial markers. As mesenchymal markers, N-cadherin, Vimentin, Snail, ZEB1, Twist, and Slug expand for mesenchymal phenotype 1. As a common gynecologic disease, endometriosis (EMS) is characterized with dynamic endometrium developing in extrauterine sites. Recently, epithelial-mesenchymal transformation is regarded as an important pathological factor in EMS 2. Wnt/β-catenin signaling is also abnormally stimulated in EMS, including promoting EMS lesions, and fibrosis 3. Notably, influence of Wnt/β-catenin signaling on epithelial-mesenchymal transformation remains unexplored in EMS.

In Chinese medicine, EMS belongs to blood stasis syndromes, especially based on the blood stasis and obstruction of uterus 4. As the famous gynecological prescription on EMS, *Foshou san* has the effects of invigorating blood and fusing stasis 5. Ferulic acid and ligustrazine are the active ingredients of *Foshou san*. Both of them join with tetrahydropalmatine to form FLT, a new Chinese herbal monomer recipe. FLT has displayed the conceivable reaction on autograft EMS 6. However, the effect of FLT is still unclear in endometrium cells and allogenic EMS.

In the study, therapeutic effect of FLT was observed *in vivo* and* in vitro*. Using endometrial cells, scratch test and transwell assays were performed after treatment with FLT. Regulations of epithelial-mesenchymal transformation and Wnt/β-catenin signaling were detected by FLT in endometrium cells and allogenic EMS.

## Materials and methods

### Animals and chemicals

ROSA^mT/mG^ mice were kindly provided by Pro. Lin Chen of Army Medical University from Jackson Lab. 18-22 g C3H mice were supplied for cross breeding (Vital River Laboratory Animal Technology, Authorization SCXK [Jing] 2016-0006, Beijing, China). 18-22 g female nude mice were used for allograft EMS (Silaike Jingda Animal, Authorization SCXK [Xiang] 2016-0002, Hunan, China). The operation of mice was approved by Care and Use of Laboratory Animals of Southwest University (Authorization 201702). Anesthesia and other necessary methods were provided to reduce suffering.

FLT were composed of 99.8% ferulic acid, 99.3% ligustrazine, and 98.1% tetrahydropalmatine (Zelang Medical Technology, Nanjing, China), with the ratio of 1:0.5:0.3. FLT were dissolved at 0.5% CMC-Na or DMSO for mice administration or endometrial cell treatment separately. The positive control selected gestrinone (Zizhu Pharmaceutical Co., Beijing, China). LiCl (Sigma-Aldrich, USA) or XAV-939 (MCE, USA) was provided as activator or inhibitor of Wnt/β-catenin pathway.

### Fluorescent allograft EMS model and treatment

Allograft operation was slightly modified from the previous studies 7. Fluorescence bilateral uteruses were collected from estrus female ROSA^mT/mG^ mice. Then, 4 mm^2^ fluorescent uteruses were transferred to subcutaneous abdomens in nude mice. After operation, nude mice were intramuscularly administrated with 2 mg·Kg^-1^ estradiol in 5 days' interval. 28 days later, ectopic lesions were captured by Fusion-FX7 imaging system (Vilber Lourmat, France). Fluorescent intensity more than 9.58E+6 were regarded as successful EMS model. According to fluorescent intensity, EMS mice were randomly into 5 groups. Firstly, EMS group was treated with CMC-Na. Secondly, there were 3 FLT groups using 90, 180, and 360 mg·Kg^-1^ FLT. Finally, another group was treated with 2 mg·Kg^-1^ gestrinone. Then, no operation mice were performed for control with same treatment as EMS group. In the end of 28 days' treatment, Evolution-Capt software (Version 18.2, Vilber Lourmat, France) was processed to investigate and analyze fluorescence intensity of ectopic lesions. Meanwhile, cubage of xenogeneic EMS was estimated with vernier caliper.

### H&E staining, E2 and PROG detection by ELISA

Eutopic and allograft endometrium were gathered from control and other 5 groups separately. Then tissues were marked with hematoxylin and eosin after fixation with paraformaldehyde. Endometrial morphology was observed in the microscope (DFC310 FX, Leica, Germany).

According to ELISA kit instruction, prepared serums were added into the equilibrated plates with dilution reagents. Then the plates were annexed with HRP-conjugate reagents, and incubated for 30 min. After incubating with chromogenic reagent, the plates were processed to stop reaction. Then absorbency was detected at 450 nm in spectrophotometer reader (Bio Tek, USA).

### Cell culture

Pro. Xiao-hong Chang of Peking University provided hEM15A cells, the human endometrial stromal cells from EMS patients 8. The endometrial carcinoma HEC1-B cells were supplied by Chinese Centre for Type Cultures Collections (Wuhan, China). hEM15A or HEC1-B cells were cultured in DMEM/F12 or MEM (Gibco, Grand Island, NY, USA) with 10% FBS (Hyclone, Shanghai, China) separately. The two cells grew in a 37 °C incubator supply of 5% CO_2_.

### Scratch wound assay

In 24-well microplate, 6-9×10^4^ endometrial cells were cultured in each well. After nearly 80% cell convergence, 1 ml pipette tip were utilized for wounded traces in plate. Treated with different doses of FLT, microplates were detected under 50 × magnification in different time points. Image software (6.0, Media Cybernetics, USA) was utilized for calculation of scratch size. Migration rate = (average scratching size in 0 h - average scratching size in 24 h)/average scratching size in 0 h × 100%.

### Transwell assay

3-5×10^4^ endometrial cells were cultured in each well with serum-free FLT medium in transwell inserts (Corning, New York, USA). The lower chambers of 24-well plate were distributed with 5% serum medium. Then plate with transwell inserts were incubated at 37 °C for 24 h. The bottom of transwell inserts were dyed with gentian violet for observation of infiltrating cells excluding surface cells. Under the microscope, 5 vision were randomly observed for infiltrating cell count.

### RNA isolation and RT-qPCR

TRIzol reagent (Invitrogen, CA, USA) was utilized RNA isolation* in vivo* and* in vitro*. Then PrimeScript™ RT Reagent Kit was purchased from Takara (China) for cDNA synthesis. SYBR™ Mix of Thermo Fisher (USA) was applied for gene expressing with 2^-∆∆CT^ calculation. Primer sequences of mRNA were produced by Shenggong Biotechnology (Shanghai, China) (Table S1). β-actin or GAPDH was performed as the control reference.

### Western blotting test

Proteins of allografts and cells were processed to western blotting following previous method 9. SDS-PAGE were used to divide the protein samples. After separation, protein of tissues and cells were delivered onto PVDF membrane. Commercial primary antibodies reacted with the protein on membranes at 4 °C overnight (Table S2). Then goat second antisubstance was provide by Multi Sciences (China) with 1:5,000 dilution. Imaging system of Tanon were utilized for chemical luminescence detection. Inner reference was selected with β-actin or β-tubulin.

### Statistical method

One-way ANOVA approach was performed for data analysis using SPSS 21.0 software. *P* values, less than 0.05 threshold, was regarded as statistical difference.

## Results

### FLT inhibited ectopic lesions, E2 and PROG levels in fluorescent allograft EMS

**Figure 1:**
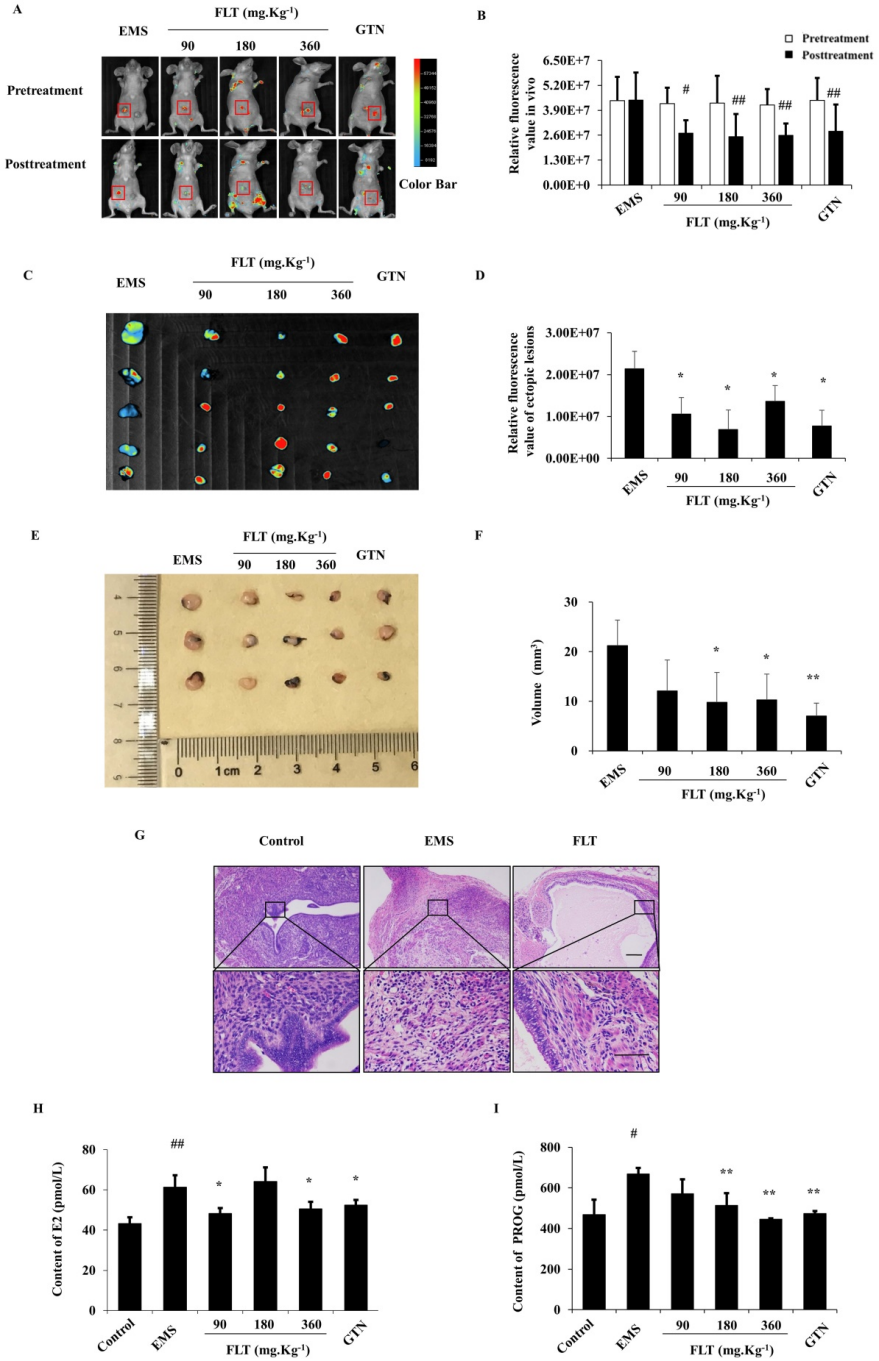
** FLT inhibited fluorescent allograft EMS. (A-D)** After treatment for 28 days, fluorescent intensity of ectopic lesions was detected* in vivo* imaging system. **(E, F)** Volume of isolated ectopic lesions were calculated by vernier caliper. **(G)** Using H&E staining, pathological changes were observed in control, EMS and 360 mg·Kg^-1^ FLT group. **(H, I)** E_2_ and PROG levels in serum were investigated by ELISA assay. #* P*< 0.05 to pretreatment, ##* P*< 0.01 to pretreatment, ∗* P*< 0.05 to EMS, ∗∗* P*< 0.01 to EMS. Columns, mean (n=5). Bars, SD. Upper scale bar=100 μm. Lower scale bar=100 μm. EMS, endometriosis; FLT, ferulic acid, ligustrazine and tetrahydropalmatine; GTN, gestrinone.

After allotransplantation for 28 days, 25 of 30 nude mice were testfied fluorescent allograft EMS with 83.33% success rate. Before treatmeant, distinct difference of fluorescent intensity were not found in EMS, FLT, and gestrinone groups. After treating for 28 days, the fluorescent intensity showed no diversity compared to pretreatment in EMS group. Using FLT, remarkable decrease of fluorescent intensity were detected in recipient nude mice (Fig. 1A, B). Then fluorescent intensity and volume of isolated ectopic issue were investigated through the second laparotomy. Compared with EMS group, FLT reduced the fluorescent intensity and volume of isolated ectopic issue (Fig. 1C-F). Meanwhile, gestrinone showed the similar effects. FLT shows the prohibition of allograft EMS growth.

Ectopic endometrium of EMS group expressed the similar structure as eutopic endometrium in control group. In ectopic endometrium, epithelial glands were surrounded by endometrial stromal tissue. Using 360 mg·Kg^-1^ FLT, pathological morphology of ectopic lesion were ameliorated, accompany with less and smaller ectopic glands, fewer microvascular and inflammatory infiltration (Fig. 1G).

EMS is regarded as a benign gynecological disease with the characteristics of estrogen and progesterone abnormity^10^. In EMS, E_2_ and PROG levels in serum were remarkably enhanced more than control group. Using FLT, E_2_ contents were remarkably diminished less than EMS group. However, PROG contents in FLT groups were significantly reduced *vs* EMS group. It was notenable that only 360 mg·Kg^-1^ FLT could decreased both E_2_ and PROG levels, same as gestrinone group (Fig. 1H, I).

### FLT prohibited epithelial-mesenchymal transformation *in vivo*

**Figure 2:**
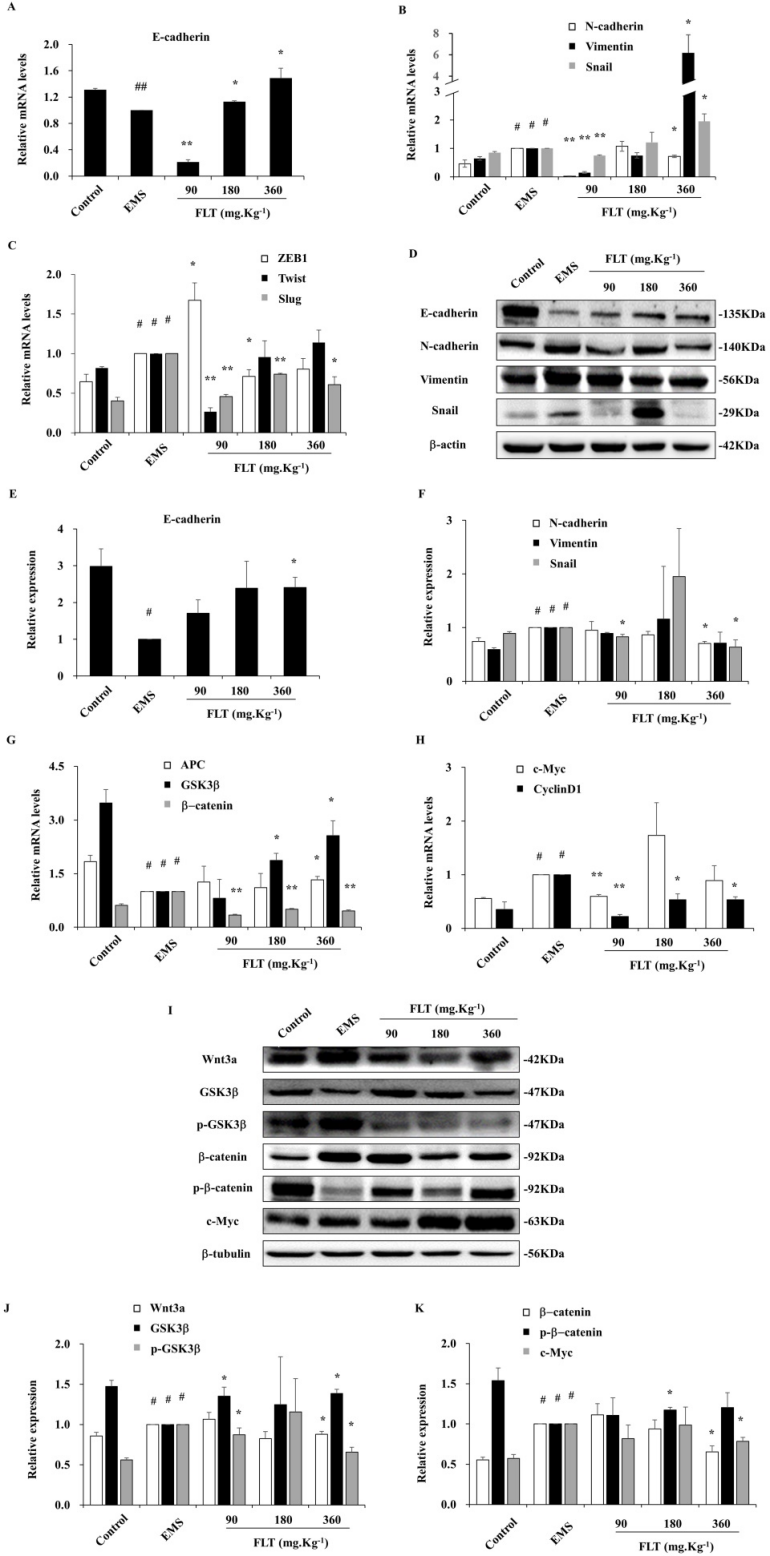
Downregulation of epithelial-mesenchymal transformation and Wnt/β-catenin pathway by FLT *in vivo*. **(A-C)** The mRNA levels of E-cadherin, N-cadherin, Vimentin, Snail, ZEB1, Twist, and Slug were detected by RT-qPCR.** (D-F)** Protein expression of E-cadherin, N-cadherin, Vimentin, and Snail were measured by western blot. **(G, H)** mRNA levels of APC, GSK3β, β-catenin, c-Myc, and CyclinD1 were detected by RT-qPCR. **(I-K)** Protein expression of Wnt3a, GSK3β, p-GSK3 β, β-catenin, p-β-catenin, and c-Myc were analyzed by western blot. # *P*< 0.05 to control, ## *P*< 0.01 to control, ∗* P*< 0.05 to EMS, ∗∗* P*< 0.01 to EMS. Columns, mean (n=3). Bars, SD. EMS, endometriosis; FLT, ferulic acid, ligustrazine and tetrahydropalmatine.

During epithelial-mesenchymal transformation, E-cadherin is considered as the epithelial marker. Meanwhile, the mesenchyme biomarkers include N-cadherin, Twist, Slug, Snail, ZEB1, and Vimentin 1. After treatment with FLT, epithelial-mesenchymal transformation markers were detected in endometrial allotransplantation. In EMS group, gene expression of E-cadherin showed remarkably decreasing by qRT-PCR (*P*<0.01). Different concentrations of FLT expressed the different effect, which were lower in 90 mg·Kg^-1^ group, and higher in 180 or 360 mg·Kg^-1^ groups (Fig. 2A). In EMS group, 6 mesenchymal mRNA levels, including N-cadherin, Twist, Slug, Snail, ZEB1, and Vimentin, obviously expanded *vs* control group. FLT obviously downregulated gene expression of N-cadherin, ZEB1, Twist and Slug, while mRNA levels of Vimentin and Snail showed the opposite changes (Fig. 2B, C). Furthermore, E-cadherin protein was remarkably reduced with raising N-cadherin, Vimentin, Snail in EMS group. FLT obviously promoted E-cadherin protein expression, accompanied by demotion of N-cadherin, Snail, Vimentin (Fig. 2D-F). The diverse results of Vimentin and Snail genes and protein were indicated that FLT might have the post-translational modification on them.

### Inhibition of Wnt/β-catenin signaling by FLT *in vivo*

Recently, EMS formation has been efficiently arrested by down-regulation of Wnt/β-catenin pathway 11. Compared with control, Wnt/β-catenin signaling was mobilized in EMS, through suppression of APC and GSK3β, enhancement of downstream β-catenin, c-Myc and CyclinD1. Using FLT, the mRNA level of APC and GSK3β obviously expanded, while β-catenin, c-Myc and CyclinD1 attenuated (Fig. 2G, H). In addition, the protein expressed similarly as genes of Wnt/β-catenin pathway. In EMS, higher levels of Wnt3a, p-GSK3β, β-catenin, and c-Myc were observed. Moreover, lower levels of GSK3β and p-β-catenin were inspected *vs* control group. FLT regulated Wnt/β-catenin pathway step by step. Firstly, FLT extremely attenuated Wnt3a. Then FLT induced the total protein of GSK3β, while decreasing p-GSK3β. Thirdly, down-regulation of β-catenin were concomitant with up-regulation of p-β-catenin. Finally, c-Myc was lessened by FLT (Fig. 2I-K).

### FLT restrain invasiveness and migration *in vitro*

**Figure 3:**
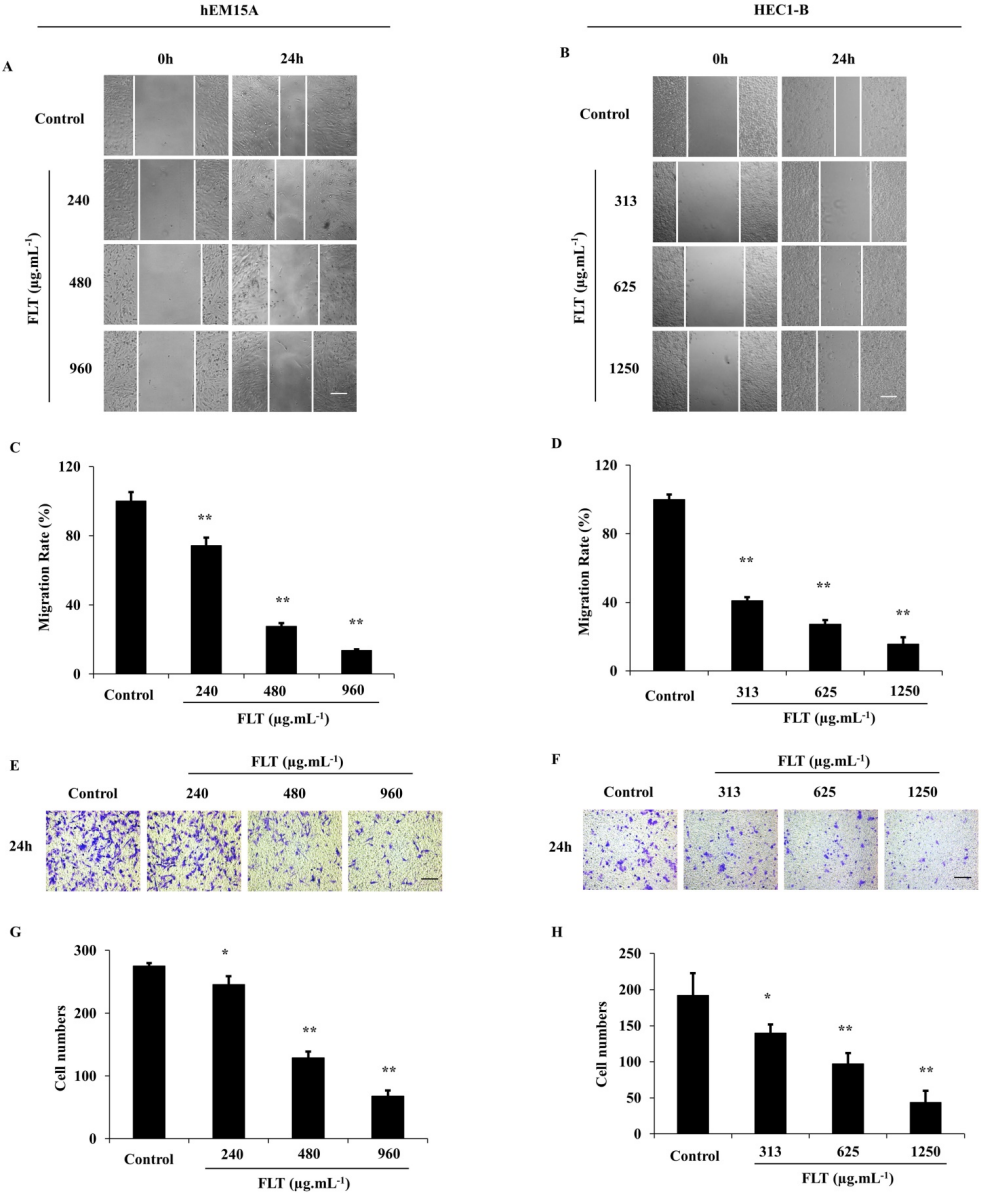
Suppression of invasion and metastasis using FLT in endometrial cells. **(A-D)** After treated with FLT, cell migration of hEM15A and HEC1-B cells was observed in scratch wound assay. **(E-H)** Transwell assay were performed to investigate migration and invasion ability. ∗* P*< 0.05 to control, ∗∗* P*< 0.01 to control. Columns, mean (n=3). Bars, SD. Scale bar=200 μm. FLT, ferulic acid, ligustrazine and tetrahydropalmatine.

After treated with FLT for 24 h, hEM15A and HEC1-B cells were prevented to cicatrize, especially in high dose (*P*<0.01) (Fig. 3A-D). Cell capacity of migration and invasiveness is investigated in transwell test. Migrating cells were significantly decreased across transwell insert membrane in FLT *vs* control (Fig. 3E-H). All above data implied that cell migration and invasion were resisted by FLT concentration-dependently.

### Suppression of epithelial-mesenchymal transformation by FLT *in vitro*

**Figure 4:**
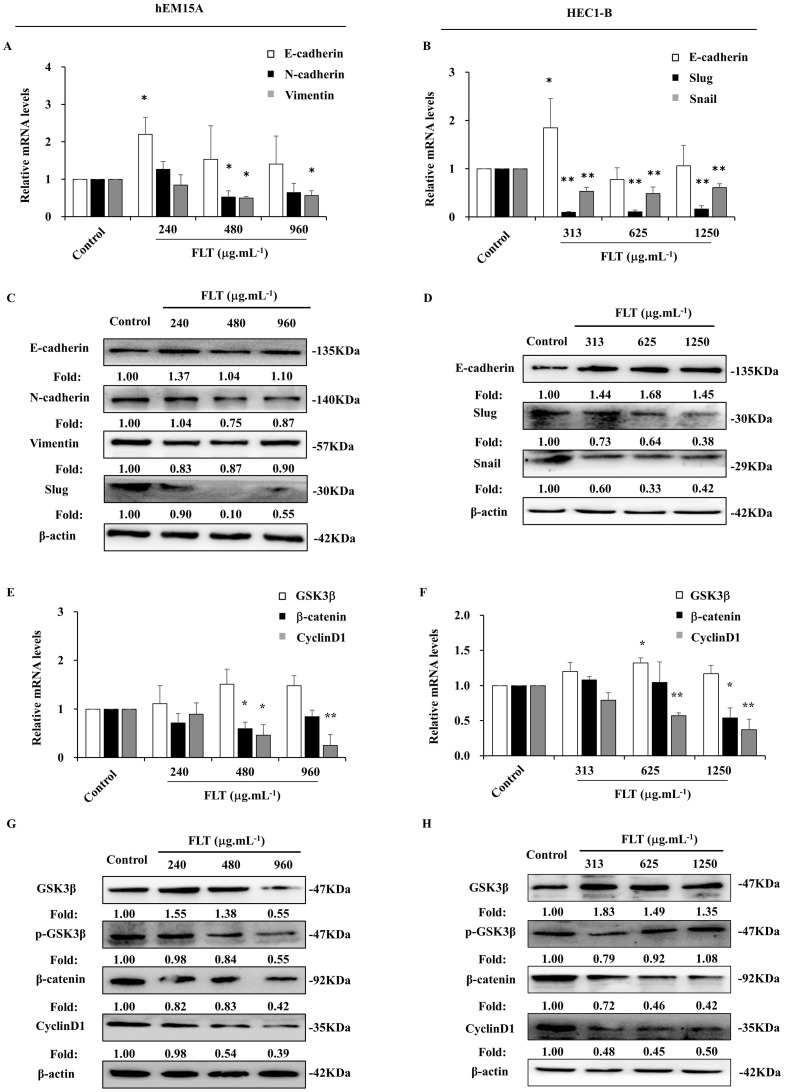
FLT downregulated epithelial-mesenchymal transformation and Wnt/β-catenin pathway in endometrial cells. **(A, B)** The mRNA levels of E-cadherin, N-cadherin, Vimentin, Snail and Slug were detected by RT-qPCR. **(C, D)** Protein levels of E-cadherin, N-cadherin, Vimentin, Slug and Snail were measured by western blot. **(E, F)** The mRNA levels of GSK3β, β-catenin and CyclinD1 were analyzed by RT-qPCR. **(G, H)** Protein levels of GSK3β, p-GSK3β, β-catenin, and CyclinD1 were observed with western blot. ∗* P*< 0.05 to control, ∗∗* P*< 0.01 to control. Columns, mean (n=3). Bars, SD. FLT, ferulic acid, ligustrazine and tetrahydropalmatine.

Epithelial-mesenchymal transformation is correlated to invasion and metastasis 12. In hEM15A cells, E-cadherin gene expression expanded after 24 h usage of 240 μg.mL^-1^ FLT. The mRNA levels of N-cadherin and Vimentin obviously attenuated in FLT groups compared to control group (Fig. 4A). Meantime, in HEC1-B cells, FLT increased mRNA level of E-cadherin, and decreased mRNA levels of Snail and Slug (Fig. 4B). Moreover, using FLT, E-cadherin protein expressed higher than control. Simultaneously, the protein of mesenchyme biomarkers was downregulated in hEM15A cells, such as N-cadherin, Slug, and Vimentin (Fig. 4C). At the same time, the protein of N-cadherin, Snail, and Slug decreased, with accumulation of E-cadherin in HEC1-B cells (Fig. 4D). The data suggested that FLT had promotion on epithelial marker, and interruption of mesenchymal markers in endometrial cells.

### Arrest of Wnt/β-catenin signaling in endometrium cells treated with FLT

After using FLT for 24 h, there was no obvious effect of FLT on GSK3β gene expression in hEM15A cells. mRNA level of β-catenin gene was remarkably reduced especially using 480 and 960 μg.mL^-1^ FLT. Treated with 480 μg.mL^-1^ FLT, the mRNA level of CyclinD1 were significantly down-regulated (Fig. 4E). Only 313 μg.mL^-1^ FLT increased the expression of GSK3β gene in HEC1-B cells, while 1250 μg.mL^-1^ FLT reduced β-catenin mRNA level. The gene expression of CyclinD1 became less in 625 and 1250 μg.mL^-1^ FLT groups than control group (Fig. 4F). Protein level of GSK3β increased in FLT group compared with control group in hEM15A cells. Simultaneously, the protein level of p-GSK3β, β-catenin, and CyclinD1 decreased (Fig. 4G). In HEC1-B cells, the protein levels of p-GSK3β, β-catenin, and CyclinD1 reduced in FLT groups, accompanied with accumulation of GSK3β (Fig. 4H).

### FLT reversed epithelial-mesenchymal transformation through regulating Wnt/β-catenin pathway

**Figure 5:**
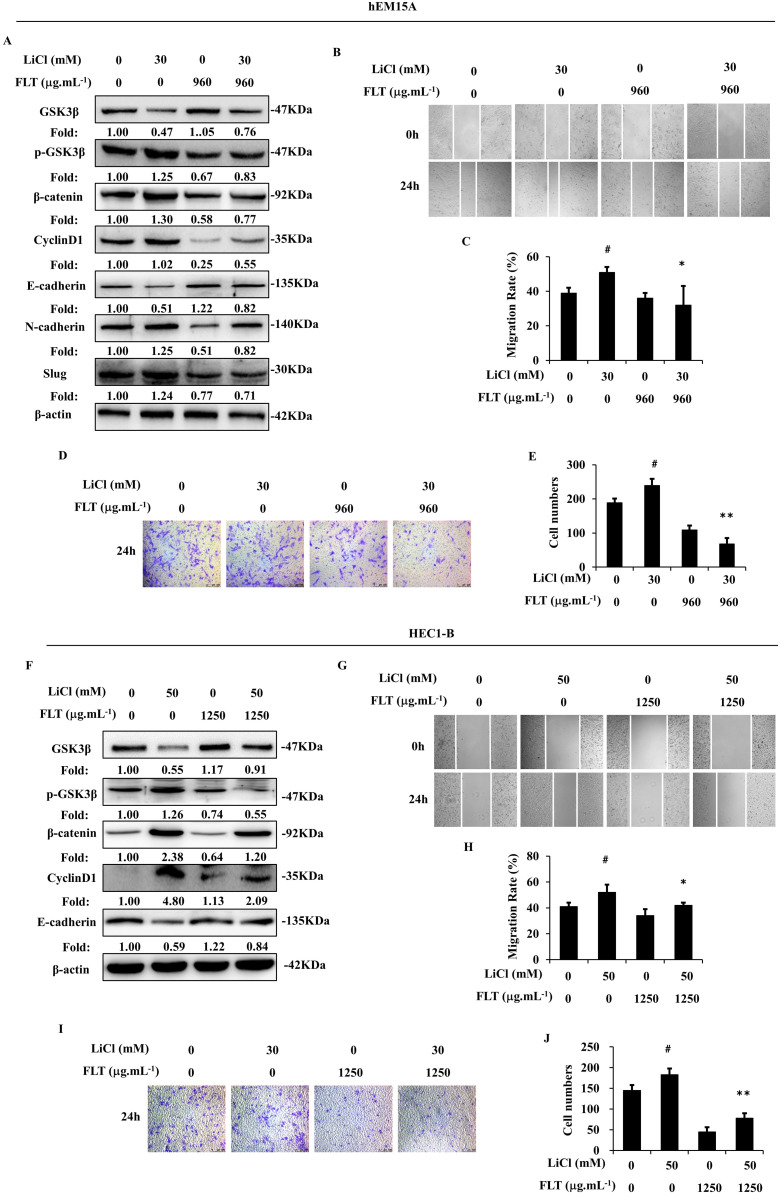
FLT reverses LiCl-induced activation of epithelial-mesenchymal transformation. **(A, F)** After using LiCl or FLT, protein levels of Wnt/β-catenin pathway and epithelial-mesenchymal transformation were observed by western blot. **(B-E, G-J)** Cell migration and invasion ability were measured by scratch wound and transwell assay in 24h. ∗* P*< 0.05 to control, ∗∗* P*< 0.01 to control. Columns, mean (n=3). Bars, SD. FLT, ferulic acid, ligustrazine and tetrahydropalmatine.

During canonical Wnt signaling, β-catenin is subsequently degraded after phosphorylated by GSK3β 13. LiCl is a special inhibitor of GSK3β, which could promote β-catenin 9. In endometrium cells, stimulation of Wnt/β-catenin signaling lead to epithelial-mesenchymal transformation induced by LiCl. Consequently, LiCl inevitably promoted cell migration and invasion. FLT reversed LiCl-activated Wnt/β-catenin pathway. Therefore, FLT restrained epithelial-mesenchymal transformation inducing by LiCl (Fig. 5A, F). At the same time, cell invasiveness and migration were inhibited by FLT in scratching and transwell assay (Fig. 5B-E, G-J). As a consequence, LiCl activated the Wnt/β-catenin pathway, promoted epithelial-mesenchymal transformation, invasiveness and migration. FLT had the antagonistic effect with LiCl.

**Figure 6:**
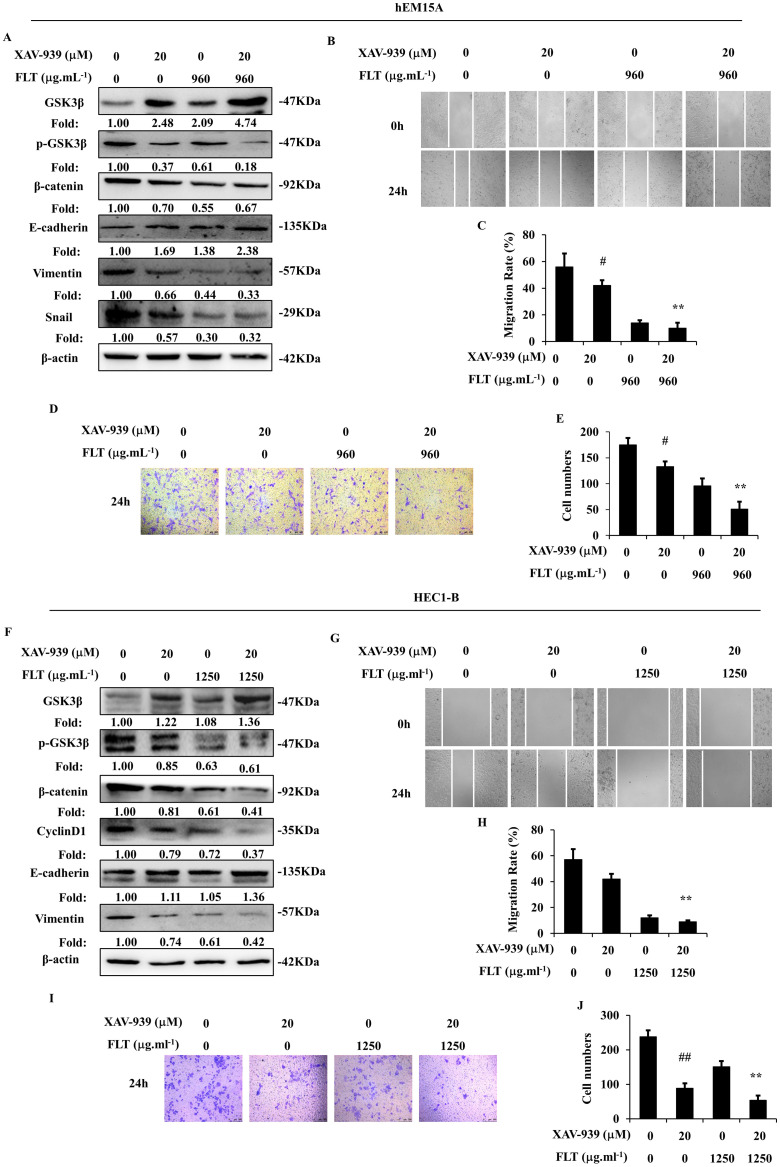
FLT enhances XAV-939-mediated attenuation of epithelial-mesenchymal transformation. **(A, F)** After using XAV-939 or FLT, protein levels of Wnt/β-catenin pathway and epithelial-mesenchymal transformation were observed by western blot. **(B-E, G-J)** Treating with XAV-939 or FLT for 24h, cell migration and invasion ability were measured by scratch wound and transwell assay. ∗* P*< 0.05 to control, ∗∗* P*< 0.01 to control. Columns, mean (n=3). Bars, SD. FLT, ferulic acid, ligustrazine and tetrahydropalmatine.

XAV-939, a tankyrase inhibitor, can increase phospho-β-catenin through inducing degradation 14. Wnt/β-catenin signaling was obviously suppressed by XAV-939 in hEM15A and HEC1-B cells. Dysfunction of Wnt/β-catenin signaling caused prohibition of epithelial-mesenchymal transformation through increasing E-cadherin and decreasing Vimentin in both cells, down-regulating Snail in hEM15A cells. Simultaneously, XAV-939 inevitably restricted invasiveness and migration in endometrial cells. FLT enhanced XAV-939-induced inactivation of Wnt/β-catenin pathway through raising GSK3β, diminishing phospho-GSK3β (Ser9), β-catenin and CyclinD1. Additionally, FLT strengthened the effect of XAV-939 on E-cadherin, N-cadherin and Snail (Fig. 6A, F). Cell migration and invasion were prevented by FLT and XAV-939 (Fig. 6B-E, G-J). In brief, XAV-939 reversed epithelial-mesenchymal transformation through repression of Wnt/β-catenin signaling, resulting in inhibition of invasion and metastasis. FLT had the synergistic effect with XAV-939.

## Discussion

In this experiment, FLT markedly inhibited fluorescent ectopic issue growth, E2 and PROG levels, ameliorated pathological change in allograft EMS model. Meanwhile, FLT prevented invasiveness and migration in endometrial cells, which was correlated to repress epithelial-mesenchymal transformation *via* Wnt/β-catenin pathway.

Nowadays, EMS has the high morbidity in reproductive age women with equivocal pathogenesis. Recognized phenomenon recovers that implantation of active endometrium cells mainly depends on invasion and metastasis in menstrual reflux hypothesis 15, 16. Epithelial-mesenchymal transformation leads to enhancement of invasion and metastasis. During this process, epithelial marker, for example E-cadherin, decreases. Mesenchymal markers, for example N-cadherin, Vimentin, ZEB1, ZEB2, Snail, Slug, and Twist increase 12, 17. Recently, Epithelial-mesenchymal transformation has been significantly elevated in EMS. Inhibition of epithelial-mesenchymal transformation reverses the progress of EMS 18-20. Consistently, in our experiment, interstitial biomarkers, including Vimentin, N-cadherin, Snail, Slug, ZEB1 and Twist enhanced, when E-cadherin as epithelial marker simultaneously attenuated in fluorescent allograft EMS mice. Interestingly, FLT restrained the growth of ectopic lesions, at the same time suppressed invasion and metastasis *in vitro*. In depth study, after treated with FLT, epithelial-mesenchymal transformation was prevented through ascendant E-cadherin, descendant interstitial biomarkers, such as N-cadherin, Vimentin, Snail, Slug, Twist, and ZEB1 *in vitro*. These data indicated the underlying mechanism of FLT on invasiveness and migration may be related to inhibit epithelial-mesenchymal transformation.

The canonical Wnt signaling is prevalent in development, differentiation, and proliferation 21. Recently, Wnt/β-catenin signaling is reported to correlate with epithelial-mesenchymal transformation in serval kinds of cancer. Wnt/β-catenin signaling activation appears that accumulation of β-catenin is transformed to nuclear. After binding with the downstream transcription factors, β-catenin triggers the target gene expression, such as Slug and Twist. Meanwhile, downregulation of E-cadherin causes less combination with β-catenin in membrane. That leads to promotion of epithelial-mesenchymal transformation 22-24. Although Wnt/β-catenin signaling dysfunction has been found in EMS, mechanism of Wnt/β-catenin signaling on epithelial-mesenchymal transformation is still await investigation. To uncover the mechanism, endometrial cells were treated with activator and inhibitor of Wnt/β-catenin signaling. Activation of Wnt/β-catenin signaling facilitated epithelial-mesenchymal transformation, invasion and metastasis. By contrast, constraint of Wnt/β-catenin pathway interrupted epithelial-mesenchymal transformation, invasion and metastasis.

Ferulic acid, ligustrazine and tetrahydropalmatine are the components of FLT, the novel Chinese herbal monomer recipe. In previous research, ferulic acid represses metastasis through withdraw epithelial-mesenchymal transformation in breast cancer 25. Furthermore, combining with other components, ferulic acid induces Wnt/β-catenin signaling, resulting in promotion of bone matrix in osteoblasts 26. Beyond that, ligustrazine restrains epithelial-myofibroblast transformation by TGF-β/Smads signaling in renal tubular epithelial cells 27. But tetrahydropalmatine on epithelial-mesenchymal transformation or Wnt/β-catenin signaling has not been reported. In our results, as the combination of ferulic acid, ligustrazine, and tetrahydropalmatine, FLT restricted Wnt/β-catenin signaling, causing downregulation of epithelial-mesenchymal transformation in allograft EMS and endometrial cells. FLT appeared the antagonistic effect with Wnt/β-catenin pathway activator; on the contrary, FLT had the synergistic effect with Wnt/β-catenin pathway inhibitor. It is worthwhile to explore other mechanism of FLT on EMS.

## Conclusion

FLT prevented invasiveness and migration in endometrium cells, and ectopic growth of allograft EMS. FLT suppressed epithelial-mesenchymal transformation through inhibiting Wnt/β-catenin signaling. These results reveal the pharmacologic mechanism for FLT further research.

## Supplementary Material

Supplementary tables.

## Abbreviations

FLT: ferulic acid, ligustrazine and tetrahydropalmatine
EMS: endometriosis
GTN: gestrinone
E2: estrogen
PROG: progesterone
H&E: hematoxylin and eosin
CMC-Na: carboxymethyl cellulose sodium
DMSO: dimethyl sulfoxide
FBS: fetal bovine serum
CO2: carbon dioxide
RT-qPCR: Reverse transcription-quantitative real-time PCR
GAPDH: glyceraldehyde-3-phosphate dehydrogenase
SDS-PAGE: sodium dodecyl sulfate-polyacrylamide gel electrophoresis
HRP: horseradish peroxidase
GSK3β: glycogen synthase kinase-3β
APC: adenomatous polyposis coli
PVDF: poplvinylidene fluoride

## References

[1] Dongre A, Weinberg RA (2019). New insights into the mechanisms of epithelial-mesenchymal transition and implications for cancer. Nat Rev Mol Cell Biol.

[2] Konrad L, Dietze R, Riaz MA, Scheiner-Bobis G, Behnke J, Horne F (2020). Epithelial-Mesenchymal Transition in Endometriosis-When Does It Happen?. J Clin Med.

[3] Shao X, Wei X (2018). FOXP1 enhances fibrosis via activating Wnt/beta-catenin signaling pathway in endometriosis. Am J Transl Res.

[4] Liao F (2000). Herbs of activating blood circulation to remove blood stasis. Clin Hemorheol Microcirc.

[5] Bai H, Li PQ, Liu J, Liu XP (2014). [Association analysis on traditional efficacy and modern research of Foshou San]. Chinese Traditional Patent Medicine.

[6] Wei JH, Zhao BX, Zhang CL, Shen BB, Zhang Y, Li CX (2019). Jiawei Foshou San Induces Apoptosis in Ectopic Endometrium Based on Systems Pharmacology, Molecular Docking, and Experimental Evidence. Evid-Based Compl Alt.

[7] Attaman JA, Stanic AK, Kim M, Lynch MP, Rueda BR, Styer AK (2014). The anti-inflammatory impact of omega-3 polyunsaturated Fatty acids during the establishment of endometriosis-like lesions. Am J Reprod Immunol.

[8] Chen Y, Li H, Cheng HY, Rui-Qiong M, Ye X, Cui H (2019). Fibrinogen alpha chain is up-regulated and affects the pathogenesis of endometriosis. Reprod Biomed Online.

[9] Chen Y, Yu DK, Zhang CX, Shang BY, He HW, Chen JJ (2015). Lidamycin Inhibits Tumor Initiating Cells of Hepatocellular Carcinoma Huh7 Through GSK3 beta/beta-Catenin Pathway. Mol Carcinogen.

[10] Barra F, Scala C, Biscaldi E, Vellone VG, Ceccaroni M, Terrone C (2018). Ureteral endometriosis: a systematic review of epidemiology, pathogenesis, diagnosis, treatment, risk of malignant transformation and fertility. Hum Reprod Update.

[11] Zhu H, Cao XX, Liu J, Hua H (2019). MicroRNA-488 inhibits endometrial glandular epithelial cell proliferation, migration, and invasion in endometriosis mice via Wnt by inhibiting FZD7. J Cell Mol Med.

[12] Pastushenko I, Blanpain C (2019). EMT Transition States during Tumor Progression and Metastasis. Trends Cell Biol.

[13] Matsuzaki S, Botchorishvili R, Pouly JL, Canis M (2014). Targeting the Wnt/beta-catenin pathway in endometriosis: a potentially effective approach for treatment and prevention. Mol Cell Ther.

[14] Lietman C, Wu B, Lechner S, Shinar A, Sehgal M, Rossomacha E (2018). Inhibition of Wnt/beta-catenin signaling ameliorates osteoarthritis in a murine model of experimental osteoarthritis. JCI Insight.

[15] Mihailovici A, Rottenstreich M, Kovel S, Wassermann I, Smorgick N, Vaknin Z (2017). Endometriosis-associated malignant transformation in abdominal surgical scar: A PRISMA-compliant systematic review. Medicine (Baltimore).

[16] Chui MH, Wang TL, Shih IM (2017). Endometriosis: benign, malignant, or something in between?. Oncotarget.

[17] Chen T, You Y, Jiang H, Wang ZZ (2017). Epithelial-mesenchymal transition (EMT): A biological process in the development, stem cell differentiation, and tumorigenesis. J Cell Physiol.

[18] Wu RF, Huang ZX, Ran J, Dai SJ, Lin DC, Ng TW (2018). Lipoxin A4 Suppresses Estrogen-Induced Epithelial-Mesenchymal Transition via ALXR-Dependent Manner in Endometriosis. Reprod Sci.

[19] Chatterjee K, Jana S, DasMahapatra P, Swarnakar S (2018). EGFR-mediated matrix metalloproteinase-7 up-regulation promotes epithelial-mesenchymal transition via ERK1-AP1 axis during ovarian endometriosis progression. FASEB J.

[20] Qi S, Yan L, Liu Z, Mu YL, Li M, Zhao X (2018). Melatonin inhibits 17beta-estradiol-induced migration, invasion and epithelial-mesenchymal transition in normal and endometriotic endometrial epithelial cells. Reprod Biol Endocrinol.

[21] Martin-Orozco E, Sanchez-Fernandez A, Ortiz-Parra I, Ayala-San Nicolas M (2019). WNT Signaling in Tumors: The Way to Evade Drugs and Immunity. Front Immunol.

[22] Pearlman RL, Montes de Oca MK, Pal HC, Afaq F (2017). Potential therapeutic targets of epithelial-mesenchymal transition in melanoma. Cancer Lett.

[23] Wu C, Zhuang Y, Jiang S, Liu S, Zhou J, Wu J (2016). Interaction between Wnt/beta-catenin pathway and microRNAs regulates epithelial-mesenchymal transition in gastric cancer (Review). Int J Oncol.

[24] Fang D, Kitamura H (2018). Cancer stem cells and epithelial-mesenchymal transition in urothelial carcinoma: Possible pathways and potential therapeutic approaches. Int J Urol.

[25] Zhang X, Lin D, Jiang R, Li H, Wan J, Li H (2016). Ferulic acid exerts antitumor activity and inhibits metastasis in breast cancer cells by regulating epithelial to mesenchymal transition. Oncol Rep.

[26] Li M, Zhang ND, Wang Y, Han T, Jiang YP, Rahman K (2016). Coordinate regulatory osteogenesis effects of icariin, timosaponin B II and ferulic acid from traditional Chinese medicine formulas on UMR-106 osteoblastic cells and osteoblasts in neonatal rat calvaria cultures. J Ethnopharmacol.

[27] Yuan XP, Liu LS, Fu Q, Wang CX (2012). Effects of ligustrazine on ureteral obstruction-induced renal tubulointerstitial fibrosis. Phytother Res.

