# Supplementary material for: Combination of Ferulic Acid, Ligustrazine and Tetrahydropalmatine attenuates Epithelial-mesenchymal Transformation via Wnt/β-catenin Pathway in Endometriosis
Source: Int J Biol Sci. 2021 Jun 11;17(10):2449–60. doi: 10.7150/ijbs.60167 (PMC8315018; doi:10.7150/ijbs.60167)
Supplement: Supplementary file 1 — Supplementary tables. [file ijbsv17p2449s1.pdf]

Table S1 primer sequences of RT-qPCR

Species	Primer name	Sequences (5'-3')
Mouse	E-cadherin-F	GGAGGAGAACGGTGGTCAAA
	E-cadherin-R	GCCACATCATTTCGAGTCAC
	N-cadherin-F	GCTTCTGGCGGCCTTGCTTCA
	N-cadherin-R	GCGTACACTGTGCCGTCCTCATCC
	Vimentin-F	CCTTGACATTGAGATTGCCA
	Vimentin-R	GTATCAACCAGAGGGAGTGA
	Twist-F	CGGGTCATGGCTAACGTG
	Twist-R	CAGCTTGCCATCTTGGAGTC
	Snail-F	ACCCACACTGGTGAGAAGCC
	Snail-R	TTGTGGAGCAAGGACATGCG
	Slug-F	GATGTGCCCTCAGGTTTGAT
	Slug-R	ACACATTGCCTTGTGTCTGC
	ZEB1-F	GATGGGGCTGCGGATGAG
	ZEB1-R	GCAGGGTGCTCTGGGTCATA
	GAPDH-F	CCTGGAGAAACCTGCCAAGTAT
	GAPDH-R	GGTCCTCAGTGTAGCCCAAGAT
	APC-F	CAAAGAAGCTGAACCTGCCAAC
	APC-R	TGCCACCCACTTTTCTAGGG
	β-catenin-F	CCTGCAGAACTCCAGAAAG
	β-catenin-R	CAAAAACATCAACGTG
	GSK3β-F	GACTCCTTTACCCTCATTACCTG
	GSK3β-R	TCTAGCATCAACTCATTTCGG
	c-Myc-F	GCGTTATTTGAAGCCTGAATTTGC
	c-Myc-R	CCTGTTAGCGAAGCTCACGTTG
	CyclinD1-F	GAACTACCTGGACCGTTTCTTG
	CyclinD1-R	AGGAAGTGTTCGATGAAATCGT
	β-actin-F	GATCATTGCTCCTCCTGAGC
	β-actin-R	ACTCCTGCTTGCTGATCCAC
Human	E-cadherin-F	CTTGGAACCCTTTATACATCTTGG
	E-cadherin-R	CCTCCCAGGTTCCAGTGATT
	N-cadherin-F	AACCCTGTCTCCCATCCAAG
	N-cadherin-R	TGCTTTCTGGCTAATGGGGA
	Vimentin-F	TGCTCCTTGGAGATGAAGCA
	Vimentin-R	CACCACTCACATCACCTCCT
	Slug-F	CGGACCCACACATTACCTTG

	Slug-R	GTGCAGGAGAGACATTCTGG
	Snail-F	ATGCCGCGCTCTTTCCTCGTC
	Snail-R	AGCAGGTGGGCCTGGTCGTAG
	β-catenin-F	GCTTCTCAAGGAGCTTACGC
	β-catenin-R	CATGTCACAGGTCGCTGATG
	GSK3β-F	GGAAGGTTGAGATGGGTGGA
	GSK3β-R	TCCAAACGATTCTCCTGCCT
	CyclinD1-F	AGATGGCACTGAATTCCCCA
	CyclinD1-R	GTCAGGACACTCAGGACCAA
	GAPDH-F	AATGGGCAGCCGTTAGGAAA
	GAPDH-R	GCCCAATACGACCAAATCAGAG

Table S2 Antibody information of Western blot

Antibody name	Dilution	Purchasing companies
rabbit anti-E-cadherin	1:1000	Cell Signaling Technology, USA
rabbit anti-N-cadherin	1:1000	Bioss Biotechnology, Beijing, China
rabbit anti-Vimentin	1:2000	Bioss Biotechnology, Beijing, China
rabbit anti-Slug	1:500	Cell Signaling Technology, USA
rabbit anti-Snail	1:500	Cell Signaling Technology, USA
rabbit anti-GSK3β	1:1000	Wanlei Biological Technology, Shenyang, China
rabbit anti-p-GSK3β	1:1000	Bioword Biotechnology, Nanjing, China
rabbit anti-β-catenin	1:1000	Wanlei Biological Technology, Shenyang, China
rabbit anti-p-β-catenin	1:1000	Sigma-Aldrich, USA
rabbit anti-c-Myc	1:1000	Wanlei Biological Technology, Shenyang, China
rabbit anti-CyclinD1	1:1000	Wanlei Biological Technology, Shenyang, China
rabbit anti-Wnt3a	1:1000	Wanlei Biological Technology, Shenyang, China
rabbit anti-β-tubulin	1:1000	Proteintech Biotechnology, Wuhan, China
rabbit anti-β-actin	1:5000	Proteintech Biotechnology, Wuhan, China
